# Systemic inflammation as a mediator between food preferences and metabolic syndrome: a cross-sectional study

**DOI:** 10.3389/fnut.2026.1761087

**Published:** 2026-05-14

**Authors:** Quanping Yan, Xueqing Liu, Yingri Zhang, Ziyuan Zhang, Yinghao Yuchi, Jian Hou, Fuwei Xie, Chongjian Wang, Ge Zhao

**Affiliations:** 1 Key Laboratory of Tobacco Chemistry, Zhengzhou Tobacco Research Institute of CNTC, Zhengzhou, China; 2 Department of Epidemiology and Biostatistics, College of Public Health, Zhengzhou University, Zhengzhou, China

**Keywords:** food preferences, mediation analysis, metabolic syndrome, systemic immune-inflammation index, systemic inflammation

## Abstract

**Backgrounds:**

Both dietary preferences and systemic inflammation are associated with metabolic syndrome (MetS); however, the role of inflammation in the association between dietary preferences and MetS remains largely unclear.

**Methods:**

A total of 33,728 participants were included from the Henan Rural Cohort Study (*n* = 39,259). Food preferences were collected using a questionnaire. Generalized linear models were used to investigate the independent and joint associations of food preferences, specifically for sour or spicy foods, and inflammatory biomarkers [such as systemic immune-inflammatory index (SII), systemic inflammatory response index (SIRI), and pan-immune-inflammation value (PIV)] with MetS prevalence. Furthermore, mediation analysis was conducted to explore the extent to which inflammation statistically accounted for the associations between food preferences and MetS.

**Results:**

Participants with a mild or moderate-to-heavy preference for sour/spicy foods had a higher risk of developing MetS compared to those who disliked these flavors, as indicated in the final model. Similarly, each unit increase in natural log-transformed SII, SIRI, and PIV values was associated with an increased risk of MetS, with corresponding odds ratios (ORs) of 1.351, 1.280, and 1.419, respectively. Individuals who had high levels of inflammation and mild-to-heavy sour/spicy food preferences exhibited the highest risk of MetS, compared to those with low levels of inflammation and a dislike for sour/spicy foods. Furthermore, the quantified statistical analysis revealed that the associations between food preferences and MetS, explained by inflammatory indicators, ranged from 5.3 to 14.6%.

**Conclusion:**

Both sour/spicy food preferences and elevated inflammatory indicators are independently and jointly associated with an increased risk of MetS. Systemic inflammation indices statistically accounted for part of the observed association, suggesting that systemic inflammation is a potential mediator of the link between food preferences and MetS.

## Introduction

Metabolic syndrome (MetS) is a cluster of risk factors including abdominal obesity, hypertension, dyslipidaemia, and raised fasting glucose (1). Kaur et al. reported that approximately 25% of adult population has MetS in the worldwide (2). The health risks of MetS are not limited to cardiovascular metabolic abnormalities (3) but also include a significantly increased the risk of obesity-related cancers, such as colon cancer and rectal cancer (4, 5). Evidence suggests that MetS significantly increases the risk of colorectal cancer, and its main drivers are thought to be persistent chronic systemic inflammation and elevated levels of insulin-like growth factor (IGF) (4, 5). MetS is also an important precursor for the development of type 2 diabetes mellitus (T2DM) (6). In addition to traditional risk factors such as high-calorie diets, daily physical activity reduction and genetic susceptibility for MetS, changes in dietary pattern are recommended as effective of prevention and control strategies for metabolic disorders (7, 8). Therefore, there is an urgent need to identify dietary factors of the metabolic disorders to implement effective measures to prevent diseases burden.

Dietary preferences are influenced by an interplay of physiological, social, and psychological factors (9). Taste preference shapes food choices and affects diet-related disease risk, originating from nutrient activation of taste receptors and neural transmission to the gustatory cortex (10). Food preferences, defined as “an individual’s reported degree of liking for specific foods and beverages, independent of actual food intake” (11), may affect adherence to dietary patterns such as the Mediterranean diet in young populations (12). Flavor perception represents a complex multisensory experience predominantly driven by olfaction through retronasal airflow during chewing or dissolution in the mouth and its impairment can compromise food identification, intake regulation, and hedonic eating experiences (13). Documented gender differences, with females often preferring sweet and sour tastes and males favoring bitter and salty flavors, may reflect evolutionary adaptations as well as sociocultural influences (14). The association between taste preferences and obesity continues to be debated in the literature (15–19). In parallel, preliminary findings connect sour taste preference with gestational diabetes (20), while spicy food liking has been linked to elevated uric acid, a factor known to worsen insulin resistance and diabetes risk (21, 22). Furthermore, spicy food intake often accompanies greater consumption of carbohydrates, salted or fatty meats, and sugary foods, possibly as a compensatory response to capsaicin-induced irritation. Notably, chronic high chili consumption (>50 g/day) may desensitize vagal afferent signaling, blunting satiety and promoting excess energy intake (15, 23). Although evidence suggests that dietary preferences are separately associated with obesity and diabetes, their specific association with MetS remains unexplored.

Systemic inflammation is hypothesized to be a central mediator connecting dietary preferences to the pathogenesis of MetS. Emerging evidence points to a bidirectional relationship, where obesity-driven inflammatory responses can disrupt taste bud homeostasis, thus linking adiposity to altered taste perception (24, 25). Furthermore, integrated biomarkers of systemic inflammation, such as the Systemic Immune-Inflammatory Index (SII) and Systemic Inflammatory Response Index (SIRI), provide enhanced prognostic information based on routine blood cell counts (26) and have demonstrated value in predicting MetS onset (27, 28). Despite these insights, the extent to which systemic inflammation explains the association between food taste preferences and MetS is not well established. This study therefore aimed to investigate the associations between food preferences and MetS and to further explore the extent to which inflammation statistically accounted for the associations between food preferences and MetS, using data from the Henan Rural Cohort Study.

## Materials and methods

### Study participants

This study utilized data from the Henan Rural Cohort, a prospective study conducted in five counties of Henan Province, China (Suiping, Yuzhou, Xinxiang, Tongxu, and Yima). At baseline (July 2015–September 2017), 39,259 participants aged 18–79 were enrolled through a multi-stage randomized cluster sampling method (29). Participants completed face-to-face interviews, along with identical questionnaires, physical examinations, and laboratory tests at both baseline and follow-up. Data were collected using a standard face-to-face questionnaire including information on demographic characteristics (age, gender and etc.), lifestyles (smoking- and drinking-status as well as physical activity), socioeconomic status (educational levels, annual family income) and personal and family history of diseases.

After excluding individuals missing information on fruits and vegetable intake (*n* = 2), total energy intake (*n* = 34), food preferences of sour (*n* = 14) and spicy (*n* = 15), personal history of diseases [diabetes (*n* = 31) and hypertension (*n* = 35)] as well as blood routine parameters such as white blood cell counts WBC (*n* = 5,287), the ratio of platelets (*n* = 5) and neutrophils (*n* = 7), waist circumference (WC, *n* = 54), body mass index (BMI, *n* = 47), 33,728 individuals were finally included for this study analysis. The proportion of missing data for the key variables was 8.5% (33,728/39,259). Given this relatively low proportion, thus we performed complete case analysis for the primary analyses.

Socioeconomic status, smoking- and drinking-status (current, former and never groups) as well as physical activity-metabolic equivalent (MET) were defined according to a previous study (30). WC was measured to the nearest 0.1 cm in light clothing. T2DM was diagnosed by either a self-reported physician diagnosis with ongoing hypoglycemic therapy (agents or insulin) or a fasting blood glucose ≥7.0 mmol/L. Hypertension was defined as systolic/diastolic blood pressure ≥140/90 mmHg or a self-reported diagnosis with current antihypertensive treatment. The SII, SIRI, and pan-immune-inflammation value (PIV) were derived from complete blood cell counts using the following formulas: SII = (platelet count × neutrophil count)/lymphocyte count, SIRI = (monocyte count × neutrophil count)/lymphocyte count and PIV = (neutrophil count × PLT count × monocyte count)/lymphocyte count (31). The study protocol was approved by the Life Sciences Ethics Review Board of Zhengzhou University prior to data collection, and written informed consent was obtained from all participants.

### Assessment of sour and spicy of food preferences

Dietary intake was assessed using a validated, semi-quantitative food frequency questionnaire (FFQ). The preferences for sour and spicy foods were evaluated separately with single-item questions: “Do you like sour/spicy food?” Responses categorized participants into four levels: Heavy, moderate, Mild, or dislike. Such single-item measures, particularly for spicy food, are well-established in prior research (32, 33). Furthermore, a pilot study (*n* = 76) confirmed the instrument’s robust reliability, demonstrating good internal consistency and high test–retest reliability, as evidenced by a high intraclass correlation coefficient (ICC) (16). In this study, the participants were classified into dislike, mild and moderate-to-heavy groups.

### Assessment of MetS

Based on the Joint Interim Societies definition and Chinese-specific WC cut-offs (34), MetS was diagnosed in participants with a WC ≥ 90/80 cm (male/female) who also met at least two of the following criteria: elevated triglycerides (≥1.7 mmol/L), low high-density lipoprotein cholesterol (HDL-C, <1.04/<1.30 mmol/L for males and females), elevated fasting glucose (≥5.6 mmol/L) or anti-diabetic medication, and elevated blood pressure (≥130/85 mmHg) or anti-hypertensive medication.

### Statistical analysis

Normally distributed continuous variables were presented as mean with standard deviation (SD). Non-normally distributed continuous variables were presented as median with interquartile range (IQR). Categorical variables were presented as number with percentage. The student’s *t* test, Mann–Whitney *U* test and Chi-square test was used to evaluate the significant differences in the selected variables between the MetS and non-MetS groups. The differences in MetS components and inflammatory indicators across preference of sour and spicy food were compared by Kruskal–Wallis test with pairwise two-sided Wilcoxon test and Benjamini–Hochberg correction.

Evaluation of model assumptions included assessment of linearity in the logit using restricted cubic splines and multi-collinearity using variance inflation factors (VIFs) in the final models. All VIF values were below 5, indicating no substantial multi-collinearity. Non-linearity of the associations between inflammatory indicators and MetS was confirmed (Supplementary Figure S1 and Supplementary Table S2), therefore, the inflammatory indicators were natural log-transformed to approximate in normal distribution. Variables were included in the modeling analysis based on either results of univariate analysis (*p* ≤ 0.10) or previous evidence indicating an association with food preferences or MetS (35–37). Logistic regression models were used to evaluate the associations of preference of sour or spicy food, inflammatory indicators with MetS risk by developing three models: Model 1 was the basic model including age and gender; Model 2 was further adjusted for education level, marital status, per capita monthly income, smoking- and drinking-status, physical activity, high-fat diet as well as fruit and vegetable intake; model 3 were further additional adjustment for the total energy intake. Furthermore, the mediation analyses were used to explore the extent to which inflammation statistically accounted for the associations between food preferences and MetS. Bootstrap was used to obtain bias-corrected confidence intervals for the direct, indirect and total effects. Prior to joint effects of food preferences and inflammation on MetS, the food preference was classified into dislike, mild-to-heavy groups and the inflammatory indicators were classified into groups by their corresponding median values as the cut-point value, the relative excess risk due to interaction (RERI) were calculated in accordance with the method described elsewhere (38).

Several sensitive analyses were performed as follows: first, these associations were further analyzed by age groups and gender. Second, the models were further adjusted for T2DM or hypertension. Third, the continuous risk score of MetS were calculated based on the method described in previous study to explore its associations with food preferences, inflammatory indicators (39). All statistical analyses were performed in the version 4.3.3 R software and the statistical significances of analysis were assigned the two-tailed *p*-value < 0.05.

## Results

### Characteristics of participants

**Figure 1:**
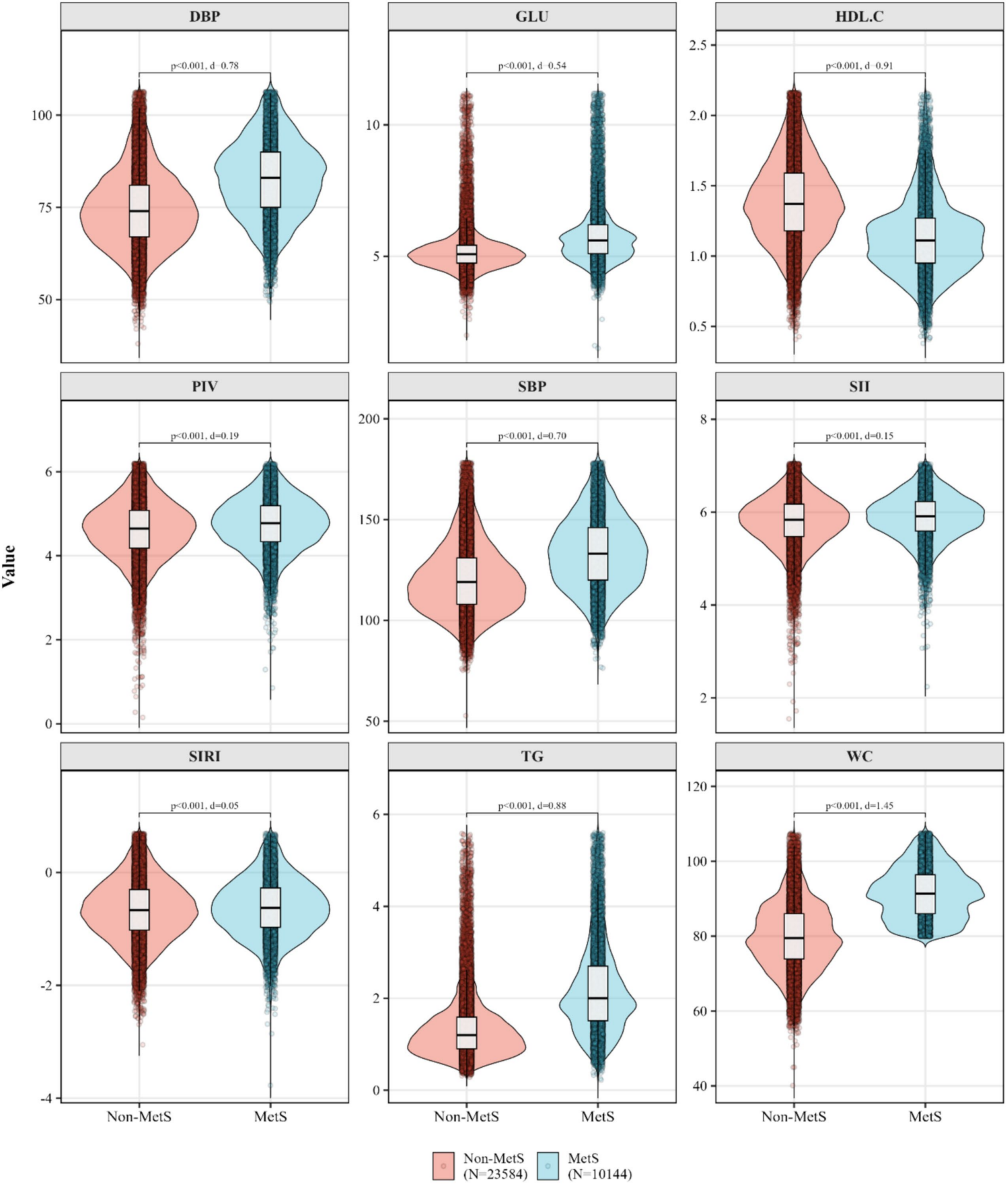
The different distributions and effect size of MetS components and inflammatory biomarkers between the non-MetS and MetS groups were analyzed using the Kruskal–Wallis test with the pairwise two-sided Wilcoxon test and Benjamini–Hochberg correction.

**Figure 2:**
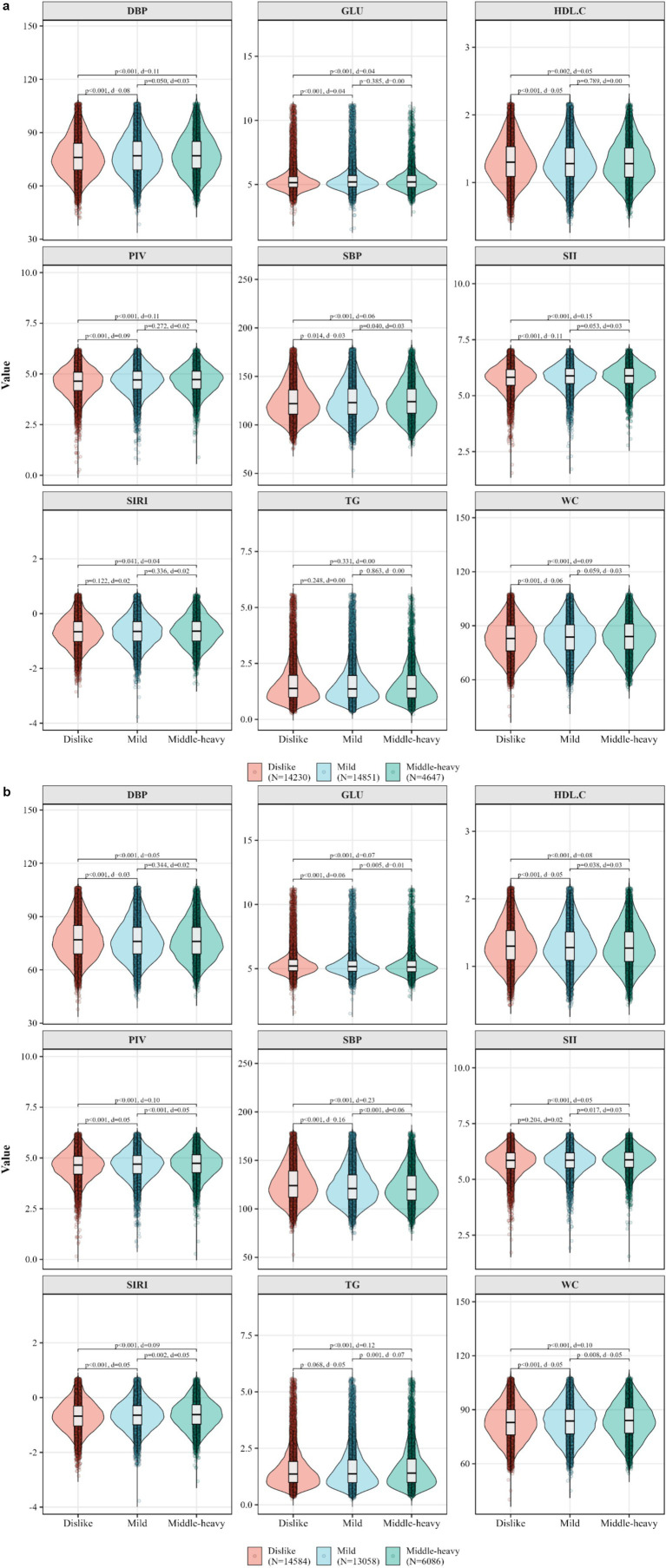
**(a,b)** The different distributions of MetS components and inflammatory indicators among different groups of sour or spicy food preferences were analyzed.

Table 1 displays that the mean (SD) age of participants with MetS was higher than the ones without MetS [57.2 (10.7) vs. 54.9 (12.7), *p* < 0.05]. The median (IQR) of SII, SIRI were found difference between MetS and non-MetS groups. Distributions of selected variables also differed between individuals with and without MetS (all *p* < 0.05). Figure 1 exhibit that the distribution of MetS components and inflammatory indicators. The median values were higher in MetS group than that in the non-MetS group, except for HDL-C. The Cohen’s d values showed a high effect size of MetS components, but a low effect size of inflammatory indicators between non-MetS and MetS groups. Figure 2a shows the distribution of MetS components and inflammatory indicators by preference of sour food. Significant differences between each two sour groups were observed for the selected variables, except that TG (all *p* < 0.05). Figure 2b shows that similar distributions by preference of spicy food, where the significant differences were found for all MetS components and inflammatory indictors between each two spicy groups. Figures 2a,b also display that most of the Cohen’s d values were less 0.2, indicating only small effects of food preference groupings on both MetS components and inflammatory indicators. These results indicates that statistically detectable differences may exist, however, the clinically modest effect sizes suggest that variation in metabolic or inflammatory status across preference groups is likely limited (Table 2).

**Table 1:** Distributions of selected variables of the participant by with and without MetS.

Variables	Non-MetS (*n* = 23,584)	MetS (*n* = 10,144)	*p*-values
Age (mean, SD)	54.51 (12.79)	57.01 (10.81)	<0.001^a^
Gender (*n*, %)			<0.001^b^
Men	10,600 (44.95)	2,956 (29.14)	
Women	12,984 (55.05)	7,188 (70.86)	
Education level (*n*, %)			<0.001^b^
Elementary school or below	9,829 (41.68)	4,910 (48.40)	
Middle school	9,721 (41.22)	3,768 (37.15)	
High school or above	4,034 (17.10)	1,466 (14.45)	
Marital status (*n*, %)			0.046^b^
Married	21,236 (90.04)	9,206 (90.75)	
Unmarried	2,348 (9.96)	938 (9.25)	
Average monthly income (*n*, %)			<0.001^b^
<500 RMB	8,397 (35.60)	3,543 (34.93)	
500–1,000 RMB	7,490 (31.76)	3,468 (34.19)	
>1,000 RMB	7,697 (32.64)	3,133 (30.89)	
Smoking status (*n*, %)			<0.001^b^
Never	16,258 (68.94)	8,127 (80.12)	
Former	2,010 (8.52)	678 (6.68)	
Current	5,316 (22.54)	1,339 (13.20)	
Drinking status (*n*, %)			<0.001^b^
Never	17,840 (75.64)	8,233 (81.16)	
Former	1,252 (5.31)	353 (3.48)	
Current	4,492 (19.05)	1,558 (15.36)	
High-fat diet (≥75 g/day, *n*, %)			<0.001^b^
Yes	4,545 (19.27)	1,774 (17.49)	
No	19,037 (80.73)	8,370 (82.51)	
Fruit and vegetable intake (≥500 g/day, *n*, %)			<0.001^b^
Yes	12,650 (53.64)	5,881 (57.98)	
No	10,934 (46.36)	4,263 (42.02)	
Physical activity-MET (hour/day, median, IQR)	18.79 (10.39)	16.94 (9.85)	<0.001^c^
T2DM (*n*, %)			<0.001^b^
Yes	1,090 (4.62)	1,940 (19.12)	
No	22,494 (95.38)	8,204 (80.88)	
Hypertension (*n*, %)			<0.001^b^
Yes	5,841 (21.6)	6,934 (57.5)	
No	21,194 (78.4)	5,120 (42.5)	
Sour			0.001^b^
Dislike	10,102 (42.83)	4,128 (40.69)	
Mild	10,271 (43.55)	4,580 (45.15)	
Moderate-to-heavy	3,211 (13.62)	1,436 (14.16)	
Spicy			0.585^b^
Dislike	10,165 (43.10)	4,419 (43.56)	
Mild	9,173 (38.90)	3,885 (38.30)	
Moderate-to-heavy	4,246 (18.00)	1,840 (18.14)	

MetS: metabolic syndrome; T2DM: type 2 diabetes mellitus.

a Student’s *t*-test was performed to compare normally distributed continuous variables between MetS and non-MetS groups.

b A Chi-square test was conducted to test the distributions of categorical variables between MetS and non-MetS groups.

c A Mann–Whitney *U* test was used to compare normally distributed continuous variables between MetS and non-MetS groups.

**Table 2:** Associations of sour or spicy taste preferences with MetS.

Variables	Model 1 (OR, 95%CI)	Model 2 (OR, 95%CI)	Model 3 (OR, 95%CI)
Sour taste preferences			
Dislike	Reference	Reference	Reference
Mild	1.146 (1.089, 1.206)	1.121 (1.064, 1.180)	1.121 (1.064, 1.180)
Moderate-to-heavy	1.182 (1.098, 1.272)	1.166 (1.083, 1.256)	1.167 (1.084, 1.257)
Spicy taste preferences			
Dislike	Reference	Reference	Reference
Mild	1.078 (1.022, 1.137)	1.081 (1.024, 1.141)	1.082 (1.025, 1.142)
Moderate-to-heavy	1.189 (1.112, 1.272)	1.206 (1.125, 1.292)	1.209 (1.128, 1.296)
Inflammatory indicators			
SII	1.366 (1.309, 1.426)	1.352 (1.294, 1.412)	1.351 (1.293, 1.411)
SIRI	1.296 (1.241, 1.354)	1.279 (1.224, 1.337)	1.280 (1.225, 1.337)
PIV	1.441 (1.392, 1.492)	1.419 (1.370, 1.470)	1.419 (1.370, 1.470)

MetS: metabolic syndrome; OR: odds ratio; 95%CI: 95% confidence interval; T2DM: type 2 diabetes mellitus; Model 1 was the basic model including age and gender; Model 2 was further adjusted for education level, marital status, per capita monthly income, smoking- and drinking-status, and physical activity; Model 3 were further additional adjustment for the total energy intake high-fat diet and fruit and vegetable intake.

### Associations of food preferences or inflammatory indicators with MetS

The generalized linear models were applied to explore associations of food preferences or inflammatory indicators with MetS. We observed that food preferences or inflammatory indicators were associated with MetS. After adjusted for age and gender, compared to the participants who disliked sour food, the estimated odd ratio (OR) and 95%CI for prevalence of MetS in participants who had a mild or moderate-to-heavy preference of sour food was 1.146 (1.089, 1.206) or 1.182 (1.098, 1.272). Compared to the participants who disliked spicy food, the estimated OR and 95%CI for prevalence of MetS in participants who had a mild or moderate-to-heavy preference of spicy food was 1.078 (1.022, 1.137) or 1.189 (1.112, 1.272). The similar results were observed in the Models 2–3. Furthermore, the estimated OR and 95% CI for prevalence of MetS in response to each-unit (natural log-transformed) increment in SII, SIRI or PIV was ranged from 1.366 to 1.441. The similar results were observed in the Models 2–3. The results of sensitive analysis show that the associations of food preference of sour food, inflammatory biomarkers with MetS were similarly across gender, only observed significantly associations of food preferences, inflammatory biomarkers with MetS among aged ≥60 participants (Supplementary Tables S2, S3). Meanwhile, the MetS score was calculated and explore its associations with food preferences, inflammatory indicators. The results show that positive associations of MetS with either food preferences or inflammatory indicators were found and the estimated *β* values were ranged from 041 to 0.137 (Supplementary Table S4).

### Combined effect of inflammation and food preferences on MetS

Table 3 shows joint associations of food preferences and inflammatory indicators with MetS. After adjusting for age and gender, education level, marital status, per capita monthly income, smoking- and drinking-status, physical activity, hypertension, T2DM and total energy intake, compared to the participants who both disliked sour food and had a low SII, participants who both had a mild-heavy preference of sour food and high SII were at a higher risk for MetS (OR: 1.076, 95%CI: 1.062, 1.091). Compared to the participants who both disliked spicy food and had a low SII, participants who both had a mild-heavy preference of spicy food and high SII were at a higher risk for MetS (OR: 1.077, 95%CI: 1.062, 1.092). The joint associations of sour or spicy food preferences and other inflammatory indicators with MetS were found the similar results. However, the additive interaction indicators (RERI) did not reach statistical significance. As shown in Supplementary Table S5, compared to participants who both disliked sour foods and were in the first tertile of inflammatory indicators, the estimated OR and 95%CI for MetS in individuals who both a mild-to-heavy preference of sour foods and were in upper tertile of the corresponding inflammatory indicators were 1.661 (1.527, 1.807), 1.576 (1.446, 1.718), or 1.996 (1.833, 2.174). Similar results were observed joint associations of taste preference of spicy and inflammatory biomarkers with MetS. Compared to individuals who both disliked spicy food and were in the first tertile of inflammatory indicators (SII, SIRI, or PIV), the estimated OR and 95%CI for MetS in individuals who both had a mild-heavy preference of spicy food and were in upper tertile of the corresponding inflammatory indicators was 1.699 (1.558, 1.852), 1.584 (1.455, 1.725) or 2.045 (1.876, 2.229). The significant multiplicative interaction of taste preferences and inflammatory indicators on MetS were found, indicating that age may modify the associations of preference of sour or spicy food, inflammatory indicators with MetS.

**Table 3:** Joint associations of sour or spicy taste preferences and inflammatory indicators with MetS^a^.

Food preferences	SII (OR, 95%CI)		SIRI (OR, 95%CI)		PIV (OR, 95%CI)	
	<Median	≥Median	<Median	≥Median	<Median	≥Median
Sour						
Dislike	Reference	1.031 (1.017, 1.045)	Reference	1.034 (1.02, 1.048)	Reference	1.037 (1.023, 1.051)
Mild-to-heavy	1.061 (1.046, 1.077)	1.076 (1.062, 1.091)	1.059 (1.044, 1.075)	1.075 (1.06, 1.089)	1.102 (1.086, 1.119)	1.111 (1.096, 1.126)
*P*_interaction_	0.091		0.053		0.002	
Spicy						
Dislike	Reference	1.033 (1.019, 1.048)	Reference	1.024 (1.01, 1.038)	Reference	1.031 (1.017, 1.046)
Mild-to-heavy	1.065 (1.05, 1.081)	1.077 (1.062, 1.092)	1.051 (1.035, 1.066)	1.070 (1.055, 1.085)	1.098 (1.082, 1.114)	1.108 (1.093, 1.124)
*P*_interaction_	0.609		0.653		0.0412	

MetS: metabolic syndrome; OR: odds ratio; 95%CI: 95% confidence interval; SII: systemic immune-inflammatory index; SIRI: systemic inflammatory response index.

a Adjusted for age, gender, education level, marital status, per capita monthly income, smoking- and drinking-status, physical activity, total energy intake, high-fat diet, and fruit and vegetable intake. All RERIs were not significant.

#### Mediation analysis of inflammation on associations of food preferences with MetS

**Figure 3:**
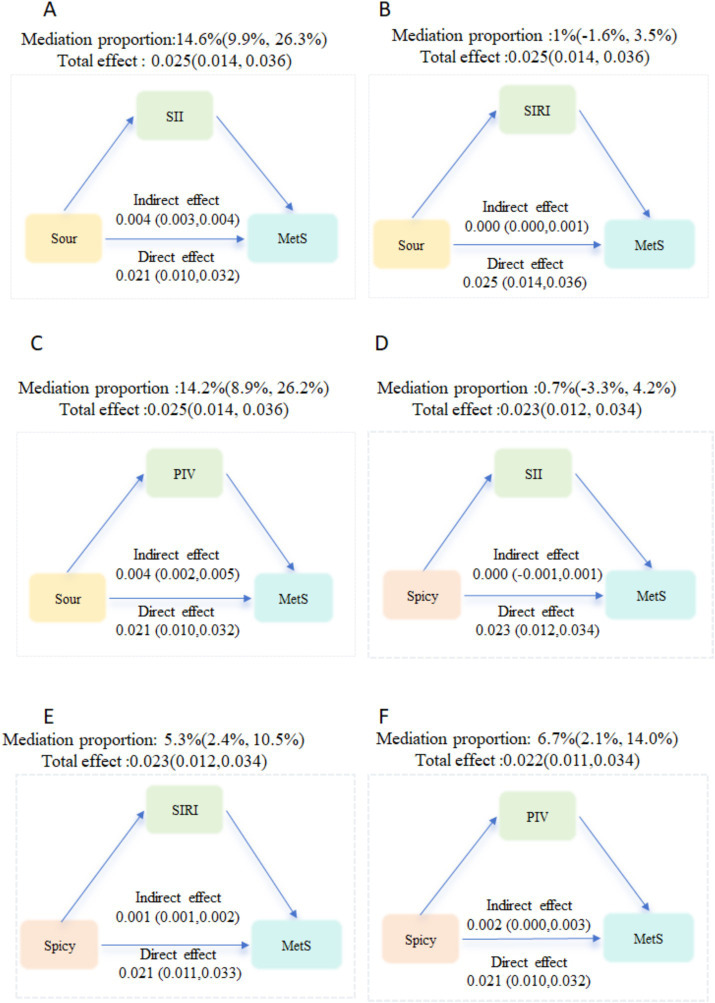
Mediation analysis was used to explore the extent to which inflammation (SII, SIRI, PIV) statistically accounted for the associations between preference of sour (A, B, C) or spicy food (D, E, F) and metabolic syndrome.

Figure 3 shows the mediation analysis was conducted to evaluate the proportion of the associations between food preferences and MetS that may be statistically explained by inflammatory indicators. After adjusting for age, gender, education level, marital status, per capita monthly income, smoking- and drinking-status, physical activity, total energy intake, high-fat diet as well as fruit and vegetable intake, the results showed that the significant direct, indirect and total effects of food preferences on MetS were found. The inflammatory biomarkers statistically accounted for 14.2 to 14.6% of the association between spicy food preference and MetS, and 5.3–6.7% of the association between sour food preference and MetS.

## Discussion

This large-scale epidemiological study yields two principal findings regarding the relationship between dietary food preferences, systemic inflammation, and MetS in a rural Chinese population. First, we identified that a moderate to heavy preference for either sour or spicy food was significantly associated with an elevated risk of MetS. Second, and more critically, our mediation analyses provide evidence that systemic inflammation, quantified by novel indices such as the SII, SIRI and PIV, may statistically explain a significant portion of the observed association between food preferences and MetS. Furthermore, the observed combined effect of food preferences and inflammation on MetS risk suggested that individuals with chronic low-grade inflammation may warrant additional attention for whom dietary tastes exert a more pronounced metabolic detriment. These findings collectively suggest that systemic inflammation is not merely an association but a potential mechanistic pathway linking food preferences to the increased risk for MetS.

The positive association between a moderate-to-heavy preference of sour food and MetS risk should be interpreted with caution. Unlike the extensively studied pathways of high sugar or fat intake, the potential mechanisms linking sour taste to MetS risk are less direct and may be confounded by the food sources that provide the sour taste. Limited evidence suggests that preference of sour food is positively related to preference for salty taste, which is a risk factor for hypertension (40, 41). Langeveld et al. reported that obese individuals consumed more energy from salt tasting food in comparison with the normal weight individuals (42). Additionally, evidence suggests that the sour taste perception is positively related to increased social jetlag (*p* = 0.002), and social jetlag has been reported to be associated with increased hypertension, obesity and metabolic dysfunction were reported (43, 44). As is well known, obesity and hypertension as the key components of MetS. This study further indicated that sour food preference is related to increased hypertension and obesity. As mentioned above evidence suggests that sour food preference in relation to increased MetS may be plausibility.

Another noteworthy finding is that spicy food preferences is associated with MetS risk. This positive association may be explained by the following reasons: First, several studies have indicated that the high intake of spicy foods significantly increases the risk of overweight and obesity (16, 23, 45). Meanwhile spicy food intake is also a risk factor for abdominal obesity among adult populations (16, 46). Moreover, a meta-analysis showed that high intake of spicy foods was related to increase the level of low-density lipoprotein cholesterol, and reduced HDL-C concentrations (45). This study also indicated that moderate-heavy spicy preference was related to increase the lipid metabolism disorders. Second, evidence indicates that spicy foods may enhance the consumption of carbohydrates, heavily salted or oily meats, and sweet foods as a means to counteract the burning sensation. Furthermore, when chili intake exceeds 50 g per day, capsaicin can desensitize vagal nerve activity, thereby blunting satiety signals and leading to increased overall food intake (15, 47). Thus, this results suggest that in a free-living population, the net effect of a sustained high spicy preference, likely embedded within a broader pattern of less healthy dietary choices, tips the balance toward an increased risk for MetS.

Although limited studies have focused on associations between taste preferences and MetS, several biologically plausible mechanisms have been suggested. Taste abnormalities associated with obesity involve multiple interacting pathways. In diet-induced obese mice, upregulation of pro-inflammatory cytokines (IL-1β, IL-6, and TNF-*α*) in taste bud cells has been observed, suggesting that diet-induced inflammation may alter the perception of dietary fats (48). Additionally, type 1 and type 2 taste receptors (T1Rs and T2Rs) may modulate inflammation through the regulation of GLP-1 secretion (49). Beyond the taste bud, dysfunction of dopamine D2 receptors (D2Rs) in the central amygdala (CeA) impairs insulin signaling and promotes compulsive eating, reinforcing a hedonic “delicious circle” driven by lipid-derived mediators and brain dopamine systems that override homeostatic needs (50, 51). Moreover, animal experiments have shown that capsaicin feeding exerts antihypertensive effects in genetically predisposed hypertensive rats and mice induced by a high-salt diet (52, 53). Taken together, these findings suggest that food preferences associated with MetS are biologically plausible; however, the potential mechanisms underlying these associations remain to be further elucidated.

Interestingly, inflammation statistically mediates the association between sour or spicy food preferences and MetS. Although effects of inflammatory biomarkers on MetS are small and may be negligible in clinical settings, these findings suggest a potential biological mechanism linking food preferences with MetS. From a public health perspective, even minor changes in inflammatory biomarkers associated with food preferences could increase a substantial burden of inflammation-related diseases. Meanwhile, inflammation as one of the key underlying mechanisms of MetS has been well documented (54). Results from the National Health and Nutrition Examination Survey showed that the inflammatory indicator SII was related to increased MetS among United States adults (27). Results from a cross-sectional study showed that SIRI was related to increased risk for MetS among Chinese rural individuals aged ≥35 years (55). Additionally, a study focused on the rural population believed that SIRI was correlated with hyperuricemia and SIRI could optimize the risk stratification of hyperuricemia (56). This study’s finding also indicated that inflammatory indicators such SII, SIRI and PIV were related to increase risk for MetS, which may further provide epidemiological evidence for the associations of novel inflammatory indicators with MetS. Previous studies have suggested that a bidirectional relationship, where obesity-driven inflammatory responses can disrupt taste bud homeostasis, thereby linking adiposity to altered taste perception (24, 25). According to the evidence mentioned above, food preferences in association with increased MetS risk may partly explain by inflammation response, and the specific mechanism need to be verified by toxicological experiments.

This study has several strengths that should be mentioned: First, the number of participants is very large. Second, addressing inflammation using multiple hematological indices, such as SII, SIRI, and PIV, rather than a single classical marker provides analytical diversity. This approach builds trust with the reader because the hypothesis is tested using multiple methods. Thirdly, the attempt to combine independent associations, combined effect, and mediation approaches within a single article is conceptually sound. Fourthly, the definition of MetS is based on an established framework. However, several limitations also should be acknowledged: First, although inflammatory indicators statistically explained the association between spicy food and sour food preferences and MetS, due to a cross-sectional study, the causal associations were not established. The findings should be confirmed by the prospective cohort studies and toxicological studies. Second, the food preference was assessed in the study through questionnaire surveys, although information of the population was collected by face-to-face interviews and all investigators underwent rigorous training on the study protocol, and standardized questionnaire administration, the potential for methodological bias such as recall bias inherent in survey-based studies cannot be fully excluded. Therefore, these limitations should be considered when interpreting the results. Third, food preference was assessed using a single question regarding subjective liking, which does not capture the actual amount, frequency, or form (e.g., cooking method) of sour or spicy food intake. Therefore, future studies incorporating comprehensive dietary assessment tools, such as food frequency questionnaires or dietary records, are needed to further explore the associations between food preferences, actual intake, and metabolic health. Fourth, although we adjusted for key sociodemographic factors, lifestyle behaviors, and total energy intake, the possibility of residual confounding remains. Unmeasured variables—including but not limited to overall dietary quality, sodium intake, medication use (e.g., statins, anti-inflammatory drugs), menopausal status, kidney function, sleep patterns, and taste disturbances—may have influenced the observed associations between food preferences, inflammation, and MetS. Additionally, we lacked data on acute infections or chronic inflammatory conditions that could affect systemic inflammation levels. While our findings suggest a potential role of inflammation in linking taste preferences to MetS, caution is warranted in interpreting these associations as causal. Future studies with comprehensive measurement of these potential confounders are needed to validate and extend our results. Finally, dietary patterns and taste preferences across different regional populations vary significantly, necessitating caution when generalizing this study’s findings.

## Conclusion

This study indicates that a middle-to-heavy preference for sour or spicy food is associated with an increased prevalence of MetS in a rural Chinese adult population. Furthermore, systemic inflammation statistically accounts for a significant proportion of these associations. Systemic inflammation may aggravate associations of food preferences with MetS, suggesting that individuals with chronic low-grade inflammation should be paid additional attention for the metabolically adverse correlates of these dietary preferences. These findings may further suggest a deeper understanding of the diet-disease relationship, with systemic inflammation as a key potential mechanistic pathway. They also may point to the potential of addressing both dietary patterns and inflammatory status for the prevention and management of MetS. Future prospective and intervention studies, using more precise measures of taste perception and dietary intake, are warranted to confirm these findings and establish causality.

## Data Availability

The raw data supporting the conclusions of this article will be made available by the authors, without undue reservation.
