# Supplementary material for: Systemic inflammation as a mediator between food preferences and metabolic syndrome: a cross-sectional study
Source: Front Nutr. 2026 May 14;13:1761087. doi: 10.3389/fnut.2026.1761087 (PMC13218083; doi:10.3389/fnut.2026.1761087)
Supplement: Supplementary file 1 [file Data_sheet_1.pdf]

Figure S1 Restricted cubic spline was used to assess the linearity of associations of inflammatory indicators with Metabolic Syndrome, after adjusted for age, gender, education level, marital status, per capita monthly income, smoking- and drinking- status, physical activity, total energy intake, high-fat diet as well as Fruit and vegetable intake.

Table S1 the VIF of associations between food preferences, inflammatory indicators with MetS in final model

**Variables**	**Sour**	**Spicy**	**SII**	**SIRI**	**PIV**
Age	1.417	1.461	1.431	1.418	1.432
Gender	2.853	2.857	2.854	2.895	2.864
Educational levels	1.38	1.379	1.376	1.376	1.376
Marital status	1.051	1.051	1.052	1.052	1.052
Average monthly income	1.098	1.097	1.097	1.097	1.098
Smoking status	2.618	2.63	2.62	2.621	2.625
Drinking status	1.899	1.911	1.894	1.893	1.895
High-fat diet	1.176	1.178	1.177	1.176	1.177
Fruit and vegetable intake	1.149	1.15	1.152	1.151	1.155
Physical activity	1.05	1.052	1.05	1.052	1.051
Total energy intake	1.425	1.427	1.427	0.2994	1.425

VIF: Variance Inflation Factor; MetS: Metabolic Syndrome

Table S2 Associations of sour or spicy taste preferences with MetS by age groups[a]

Variables	age<60 (OR, 95%CI)	age≥60 (OR, 95%CI)	$P_{interaction}$
Preference of sour food			
Unlike	Reference	Reference	
Mild	0.952 (0.889, 1.019)	1.389 (1.282, 1.505)	0.008
Middle-heavy	1.099 (0.998, 1.211)	1.301 (1.158, 1.461)	
Preference of spicy food			
Unlike	Reference	Reference	
Mild	1.032 (0.960, 1.109)	1.060 (0.976, 1.150)	0.036
Middle-heavy	1.144 (1.050, 1.247)	1.131 (1.005, 1.274)	
Inflammatory indicators			
SII	1.313 (1.237, 1.393)	1.384 (1.297, 1.477)	0.951
SIRI	1.292 (1.219, 1.370)	1.277 (1.194, 1.366)	<0.001
PIV	1.390 (1.326, 1.457)	1.431 (1.356, 1.509)	0.012

MetS: metabolic syndrome; OR: odd ratio; 95%CI: 95% confidence interval; SII: systemic immune-inflammatory index; SIRI: systemic inflammatory response index; PIV: Pan-immune-inflammation value; [a]adjusted for age, gender, education level, marital status, per capita monthly income, smoking- and drinking- status, physical activity, total energy intake, high-fat diet as well as Fruit and vegetable intake.

Table S3 Associations of sour or spicy taste preferences with MetS by gender[a]

Variables	Males (OR, 95%CI)	Females (OR, 95%CI)	Pinteraction
Preference of sour food			
Unlike	Reference	Reference	
Mild	1.100 (1.003, 1.207)	1.154 (1.083, 1.230)	0.880
Middle-heavy	1.188 (1.050, 1.343)	1.212 (1.102, 1.333)	
Preference of spicy food			
Unlike	Reference	Reference	
Mild	1.096 (0.994, 1.208)	1.094 (1.024, 1.169)	<0.001
Middle-heavy	1.240 (1.103, 1.393)	1.214 (1.112, 1.325)	
Inflammatory biomarkers			
SII	1.290 (1.196, 1.393)	1.438 (1.362, 1.519)	0.049
SIRI	1.295 (1.199, 1.397)	1.325 (1.254, 1.400)	0.067
PIV	1.409 (1.324, 1.499)	1.465 (1.402, 1.531)	0.058

MetS: metabolic syndrome; OR: odd ratio; 95%CI: 95% confidence interval; SII: systemic immune-inflammatory index; SIRI: systemic inflammatory response index; PIV: Pan-immune-inflammation value; [a]adjusted for age, gender, education level, marital status, per capita monthly income, smoking- and drinking- status, physical activity, total energy intake, high-fat diet as well as Fruit and vegetable intake.

Table S4 Associations of sour or spicy taste preferences with MetS score

Variables	Model 1 (β, 95%CI)	Model 2 (β, 95%CI)	Model 3 (β, 95%CI)
Preference of sour food			
Dislike	Reference	Reference	Reference
Mild	0.049 (0.029, 0.070)	0.041 (0.021, 0.061)	0.041 (0.021, 0.061)
Middle-heavy	0.047 (0.018, 0.077)	0.043 (0.014, 0.072)	0.043 (0.014, 0.072)
Preference of spicy food			
Dislike	Reference	Reference	Reference
Mild	0.051 (0.029, 0.072)	0.051 (0.030, 0.072)	0.051 (0.030, 0.072)
Middle-heavy	0.092 (0.065, 0.119)	0.098 (0.071, 0.125)	0.097 (0.070, 0.124)
Inflammatory indicators			
SII	0.110 (0.094, 0.127)	0.107 (0.090, 0.124)	0.108 (0.091, 0.125)
SIRI	0.098 (0.081, 0.115)	0.092 (0.074, 0.109)	0.092 (0.074, 0.109)
PIV	0.141 (0.128, 0.154)	0.136 (0.123, 0.150)	0.137 (0.123, 0.150)

MetS: metabolic syndrome; β: beta coefficient; 95%CI: 95% confidence interval; SII: systemic immune-inflammatory index; SIRI: systemic inflammatory response index; PIV: Pan-immune-inflammation value; Model 1 was the basic model including age and gender; Model 2 was further adjusted for education level, marital status, per capita monthly income, smoking- and drinking- status, physical activity, high-fat diet as well as fruit and vegetable intake; model 3 were further additional adjustment for the total energy intake.

Table S5 Joint associations of preference of sour or spicy food and inflammatory indicators with MetS[a]

Food preferences	SII (OR, 95%CI)			SIRI (OR, 95%CI)			PIV (OR, 95%CI))		
	< 284.05	284.05-441.00	≥ 441.00	< 0.42	0.42-0.67	≥ 0.664	< 81.56	81.56-145.51	≥ 145.51
Sour									
Unlike	Reference	1.422(1.297,1.558)	1.577(1.438,1.729)	Reference	1.255(1.145,1.375)	1.475(1.345,1.617)	Reference	1.462(1.333,1.604)	1.909(1.741,2.094)
Mild-heavy	1.169 (1.071 ,1.276)	1.607(1.477,1.749)	1.661(1.527,1.807)	1.173(1.077,1.277)	1.452(1.333,1.58)	1.576(1.446,1.718)	1.204(1.103,1.315)	1.626(1.493,1.771)	1.996(1.833,2.174)
$P_{interaction}$		0.060			0.121			0.019	
Spicy									
Unlike	Reference	1.458(1.332,1.596)	1.581(1.445,1.73)	Reference	1.302(1.191,1.422)	1.429(1.306,1.565)	Reference	1.526(1.394,1.669)	1.851(1.69,2.027)
Mild-heavy	1.189(1.089,1.299)	1.617(1.483,1.763)	1.699(1.558,1.852)	1.161(1.066,1.265)	1.392(1.279,1.515)	1.584(1.455,1.725)	1.207(1.105,1.319)	1.581(1.45,1.725)	2.045(1.876,2.229)
$P_{interaction}$		0.091			0.411			0.160	

MetS: metabolic syndrome; OR: odd ratio; 95%CI: 95% confidence interval; SII: systemic immune-inflammatory index; SIRI: systemic inflammatory response index; T2DM: type 2 diabetes mellitus; [a] adjusted for age, gender, education level, marital status, per capita monthly income, smoking- and drinking- status, physical activity, total energy intake, high-fat diet as well as fruit and vegetable intake.

Figure S1

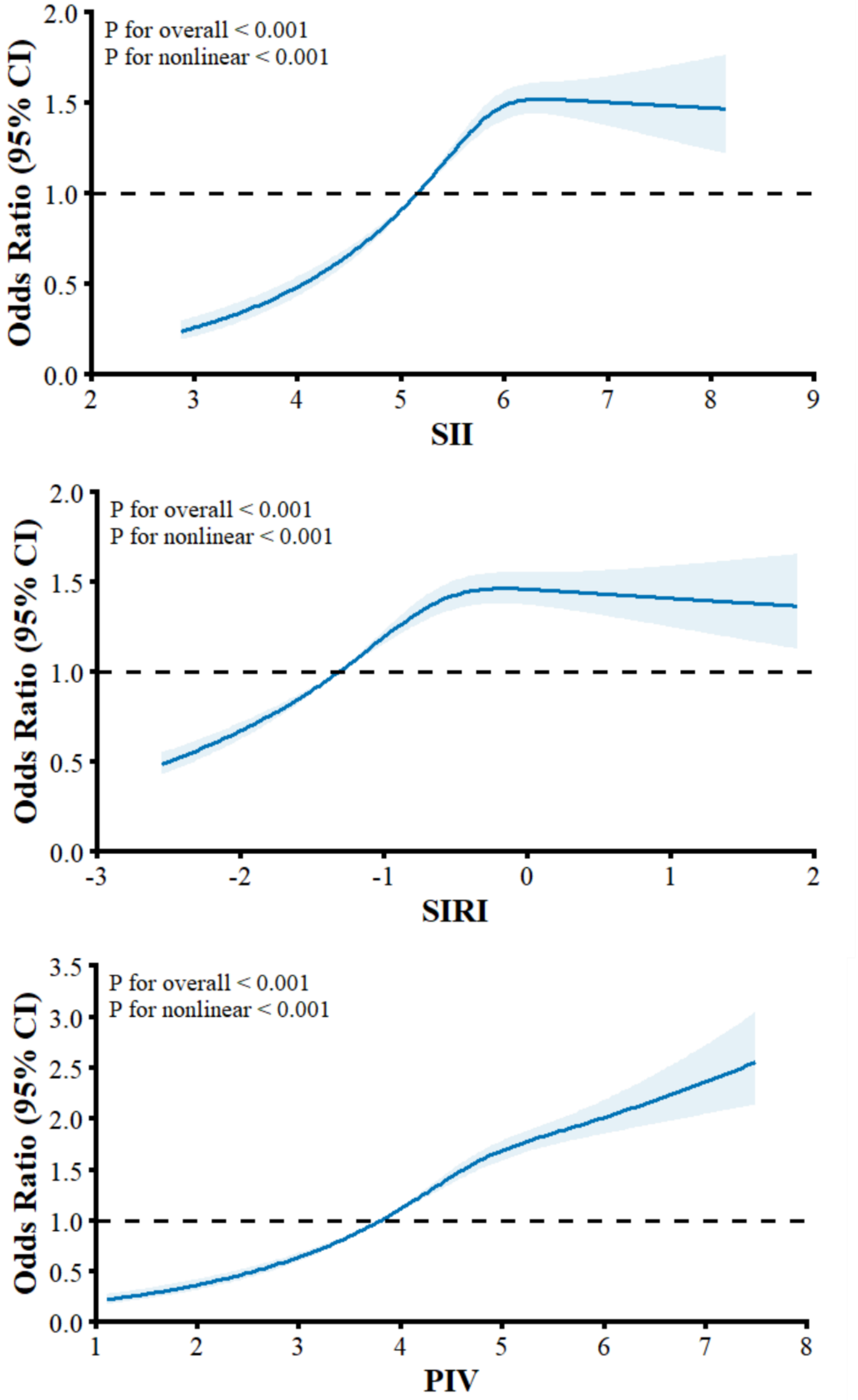
P for overall < 0.001
P for nonlinear < 0.001
Odds Ratio (95% CI)
SII
P for overall < 0.001
P for nonlinear < 0.001
Odds Ratio (95% CI)
SIRI
P for overall < 0.001
P for nonlinear < 0.001
Odds Ratio (95% CI)
PIV
